# Preventing and Reducing Coercive Measures—An Evaluation of the Implementation of the Safewards Model in Two Locked Wards in Germany

**DOI:** 10.3389/fpsyt.2019.00340

**Published:** 2019-05-24

**Authors:** Johanna Baumgardt, Dorothea Jäckel, Heike Helber-Böhlen, Nicole Stiehm, Karin Morgenstern, Andre Voigt, Enrico Schöppe, Ann-Kathrin Mc Cutcheon, Edwin Emilio Velasquez Lecca, Michael Löhr, Michael Schulz, Andreas Bechdolf, Stefan Weinmann

**Affiliations:** ^1^Department of Psychiatry, Psychotherapy and Psychosomatic Medicine, Vivantes Hospital Am Urban und Vivantes Hospital im Friedrichshain, Charité–Universitätsmedizin Berlin, Berlin, Germany; ^2^Department of Psychiatry and Psychotherapy, Center for Psychosocial Medicine, University Medical Center Hamburg-Eppendorf, Hamburg, Germany; ^3^Landschaftsverband Westfalen-Lippe, Hospital Gütersloh, Gütersloh, Germany; ^4^Diakonie University of Applied Sciences, Bielefeld, Germany; ^5^ORYGEN, National Center of Excellence of Youth Mental Health, University of Melbourne, Melbourne, VIC, Australia; ^6^Department for Psychiatry and Psychotherapy, University Hospital Cologne, Cologne, Germany; ^7^University Psychiatric Hospital Basel, Basel, Switzerland

**Keywords:** Safewards Model, conflict, coercive measures, acute psychiatric care, inpatient treatment, locked ward

## Abstract

**Introduction:**

Aggression and violence are highly complex problems in acute psychiatry that often lead to the coercive interventions. The Safewards Model is an evidence-informed conflict-reduction strategy to prevent and reduce such incidents. The aim of this study was to evaluate the implementation of this model with regard to coercive interventions in inpatient care.

**Materials and Methods:**

We evaluated outcomes of the implementation of the Safewards Model in two locked psychiatric wards in Germany. Frequency and duration of coercive interventions applied during a period of 11 weeks before and 11 weeks after the implementation period were assessed through routine data. Fidelity to the Safewards Model was assessed by the Organization Fidelity Checklist.

**Results:**

Fidelity to the Safewards Model was high in both wards. The overall use of coercive measures differed significantly between wards [case-wise: χ^2^ (1, *n* = 250) = 35.34, *p* ≤ 0.001; patient-wise: χ^2^ (1, *n* = 103) = 21.45, *p* ≤ 0.001] and decreased post-implementation. In one ward, the number of patients exposed to coercive interventions in relation to the overall number of Patients decreased significantly [χ^2^ (1, 281) = 6.40, *p* = 0.01]. Furthermore, the mean duration of coercive interventions overall declined significantly [*U*(55,21) = −2.142, *p* = 0.032] with an effect size of Cohen’s *d* = −0.282 (95% CI: −0.787, 0.222) in that ward. Both aspects declined as well in the other ward, but not significantly.

**Discussion:**

Results indicate that the implementation of the Safewards interventions according to the model in acute psychiatric care can reduce coercive measures. They also show the role of enabling factors as well as of obstacles for the implementation process.

## Introduction

Managing conflict and violent situations such as self-harm, drug abuse, physical aggression, verbal abuse, or aggressive behavior towards others is part of acute inpatient psychiatric care. It has become a major focus for staff interventions in such units (1, 2). Coercive interventions are often used to contain such situations, even though they may cause harm to patients and staff (3). Rates of coercive interventions vary between individual units and across countries. A previous study suggests that coercive measures are used in between 21% and 59% of individuals admitted to psychiatric hospitals across various European countries (4). Types of coercive intervention used varies between countries depending on the national psychiatric legislations (5–9). Coercive measures are associated with longer duration of inpatient treatment and forced medication seems to have a significant impact on patient disapproval of treatment (10).

Coercive interventions are considered a violation of human rights according to the United Nations Convention on the Rights of Persons with Disabilities (11). They should only be applied in case of emergency, as last option if other measures failed, and under strict conditions (12). Their ethical and clinical appropriateness is a priority in many countries aiming to reduce their use in mental health care (3, 13).

Against this background, the implementation of strategies that prevent and reduce coercive interventions is widely recommended and requested (14, 15). Nevertheless, such strategies are only partially realized so far (16–18). This might—*inter alia*—result from the complexity of the issue. This complexity is, e.g., outlined by a recent systematic review that revealed six key components in coercive measure reduction programs: 1) leadership, 2) training, 3) post-seclusion and/or restraint review, 4) patient involvement, 5) prevention tools, and 6) therapeutic environment (19). According to current knowledge, complex interventions appear to be particularly effective in reducing coercion measures if they contain various components. One of the most recently developed programs containing all those components is the Safewards Model (20). This conflict-reducing strategy accounts as an evidence-based complex intervention in psychiatric care. Its great merit is its strong theoretical basis and the conclusiveness with which it has been assessed. The Safewards Model was developed in the United Kingdom and has been translated into several languages (1, 21). It combines empirical evidence regarding aggression, flight behavior, and containment with new thoughts on preventing aggression and violence (22–27). The theoretical framework of the model is particularly characterized by the complex interplay between conflict and containment (1). Thus, the Safewards Model has a broader perspective than explanatory models that look at these aspects separately (28–30). Furthermore, it distinguishes between the cause, the triggers, and the actual occurrence of a conflict. Its explanatory framework outlines situations of tension that develop within regular procedures in acute psychiatry (31). For the first time, a theoretical model on conflict and containment includes the influence of patient interactions and regulatory frameworks as well as external conditions and patient behavior. Moreover, the Safewards Model advocates the need for more safety for patients and staff from the highest hierarchical level (20). It is grounded in recovery principles and identifies six key domains that influence conflict and containment: patient community, patient characteristics, regulatory framework, staff team, physical environment, and factors from outside the hospital. By this multi-perspective approach, it offers several starting points to prevent conflict and coercive interventions in acute psychiatry (31).

The core of the Safewards Model consists of 10 interrelated interventions (2, 31):

**Clear Mutual Expectations:** Staff holds regular meetings with patients to discuss expectations of each other’s behavior. A final set of expectations is printed on a poster and displayed on the ward visible for patients and staff.

**Soft Words:** About 100 statements are provided to staff to advise them on how to speak to patients around “flashpoints” such as when staff have to say “no” to a patient, when staff has to ask a patient to do something that they don’t want to do, or when staff has to ask the patient to stop doing something that they should not do.

**Talk Down:** Staff is taught a process for de-escalation and how to integrate this into everyday practice.

**Positive Words:** Staff is encouraged to say something positive about the patients during their handover that is supported by a positive psychological explanation of observed behavior.

**Bad News Mitigation:** Staff is taught specific techniques to assist them in delivering “bad” news to patients.

**Know Each Other:** Staff provides non-controversial information about themselves such as hobbies, interests, etc. This is made visible or rather available to patients and forms the basis for better interactions with staff.

**Mutual Help Meeting:** Each morning or several times a week, staff holds a patient meeting to identify ways that patients can help each other during the ensuing 2 days.

**Calm Down Methods:** Staff is taught specific activities (“skills”) to assist patients to calm down when they are tense or agitated.

**Reassurance:** Following the occurrence of an adverse event or an anxiety-provoking incident on the ward, staff should talk to other patients individually or in groups to provide information on what has happened and reassure patients.

**Discharge Messages:** On the day of their discharge, patients are invited to write a brief card for display on a special notice board. The cards relate to what they liked about their stay and to positive thoughts about the future. The aim of these cards is to help recently admitted patients to reduce negative feelings and concerns about hopelessness.

These 10 interventions are taught to and later carried out by all professional groups in the ward. The main goal of the training is to prevent and detect conflict situations at an early stage and respond with specific verbal as well as non-verbal communication. Thereby, the rate of conflict shall be reduced. At the same time, participation, appreciation, hope, and empowerment shall be reinforced.

There are promising research findings indicating a positive effect of the Safewards Model on conflict and containment in acute inpatient units in English-speaking countries (2, 32, 33). For example, a large-scale randomized controlled trial in the United Kingdom found a reduction in conflict by 15% and a reduction in containment by 25% in intervention versus control wards (34). However, confidence intervals were rather broad, suggesting varying success with implementation of the Safewards Model or difficulties with regard to model fidelity (35). Poor adherence was also discussed as a limitation in a study conducted in forensic units that did not find positive effects after implementing the Safewards Model (36). High fidelity was measured in an Australian study that found seclusion rates to be reduced by 36% after the Safewards implementation in psychiatric inpatient units (33). Further empirical evidence for reducing conflict and containment in psychiatry was found regarding several multi-perspective conflict-reduction interventions focusing on leadership, staff, and patient level (18, 37, 38). Richter and Needham (18), e.g., showed that participating in de-escalation and aggression management staff training can lead to better knowledge and a more precise documentation of conflicts and containment. Furthermore, their review outlined that studies using outcomes close to the intervention, e.g., increase of knowledge and confidence, showed almost homogeneous positive results. However, results from studies investigating a combination of defense and de-escalation techniques were heterogeneous. These studies found positive effects as well as negative or no effects of such staff training on the rate of conflicts. With regard to the latter findings, Richter and Needham (18) point out that the role of change in documentation of aggressive events is uncertain since staff training increases the willingness to register such events.

To our knowledge, there is no evaluation of an implementation of the Safewards Model in acute psychiatric inpatient units or rather locked wards in the German health care context so far. Thus, we aimed at transferring research findings into clinical practice as well as examining underlying factors that might promote or hinder this process. This procedure is closely related to methods of implementation of science and shall facilitate the transfer of evidence-based clinical–psychological into acute psychiatric mental health care (39, 40). We hypothesized that changes in the ward through the implementation of the Safewards Model would reduce coercive interventions. Thus, we evaluated the implementation process with regard to the use of mechanical restraint, forced medication, and limitation of freedom of movement. This procedure aimed at closing another research gap since the majority of evaluations regarding the reduction of coercive interventions assessed the impact only on mechanical restraint (41). Furthermore, considerable differences in national laws and practices as well as the lack of studies reporting on the effects on other types of coercion affect the transferability of these results to other countries. For example, in the United Kingdom, mechanical restraint is not routinely used, and in Australia, only seclusion is used in mental health care. In Germany, on the other hand, mechanical restraint, forced medication, limitation of freedom of movement, and seclusion are used in different hospitals. Therefore, we assessed the use of different coercive measures and reported them individually.

## Materials and Methods

The Safewards Model was implemented in two locked wards in the Department of Psychiatry, Psychotherapy and Psychosomatic Medicine at Vivantes Hospital Am Urban in Berlin. Vivantes Hospital Am Urban has one central emergency department, 11 medical and surgical departments and one psychiatric department. The psychiatric department comprises two outpatient departments and 614 beds including 174 psychiatric inpatient beds and 50 day clinic beds. The hospital’s catchment area is Berlin’s inner-city district Friedrichshain-Kreuzberg with approximately 280,000 residents.

### Implementation and Evaluation of the Safewards Model

In the implementation and evaluation process of the Safewards Model, experienced hospital staff as well as experienced external experts were involved. Hospital staff was familiar with the implementation of new interventions through continuous work on psychiatric projects in the hospital. Michael Löhr (ML) and Michael Schulz (MS), from the University of Bielefeld, guided the process as external experts. They had translated the Safewards Model into the German language and adapted it to the German care system. Furthermore, ML and MS play a central role in establishing and implementing the Safewards Model in Germany (42–45). In addition, they have a wide range of experience in dealing with coercion (46–48).

The framework that guided the implementation concept developed by MS and ML consisted of three parts: 1) step-by-step plan for developing the implementation intervention, 2) implementation, and 3) evaluation of the implementation.

### (1) Step-by-Step Plan for Developing the Implementation Intervention

First, a five-step implementation plan was developed following Skolarus and Sales (49) while considering relevant literature of the Safewards concept:

1. A review of current practice regarding violence and coercion in the hospital was conducted. This revealed problems in both locked wards.
2. Literature analyses and exchanges with experts resulted in the selection of the Safewards Model as an evidence-based practice for improving the situation.
3. Barriers that could hinder the implementation of the Safewards Model were identified.
4. Concrete actions were taken to address possible barriers. Among others, the implementation opted for a whole-team approach with participation and motivation of all professions and the entire staff of the ward. It was decided that all professional groups should participate in the training. Through participative sessions, employees’ motivation was promoted.

### (2) Implementation

A wide range of essential aspects had to be considered during the implementation, e.g., approval and integration of executive management or nomination and definition of tasks of operative project management. For this purpose, the Safewards preparation checklist was applied throughout the whole process (45). Initially, a steering group with the director of the department, the nursing director, the consultant psychiatrists responsible for the ward, and a project manager for each ward was established. The manualized 10 Safewards team interventions were introduced within a standardized all-day workshop carried out by ML and MS with all staff members of the two wards (50). Afterwards, executive staff members were asked if they were interested in dealing with the concept in detail. Each interested staff member was assigned to one of the interventions as an “expertise multiplier” or rather “Safewards champion.” The person holding this position was responsible for planning, organizing, and implementing the corresponding intervention. According to Houser et al. (51), such a nomination is an effective strategy for information transfer in an implementation process. After the nomination, assigned executive staff members developed a concept as well as working material for implementing “their” intervention in the ward. These were based on the manual of the Safewards resource kit from the official Safewards web page (http://www.safewards.net/de/). Issues dealt with were, e.g., educational aspects regarding how to give the team an understanding of the intervention, how the intervention can be integrated into daily work routines, or which resources were required such as rooms, time, money, and equipment. The status of the implementation and the material used were presented in a separate workshop. The workshop was led by ML and MS who provided feedback and suggestions for improvement of the ideas presented. Cultural and local adaptation was developed in this process in close exchange between staff and external experts. The aim of this process was to facilitate a successful implementation while maintaining the main profile of the intervention. Some of the interventions had to be adapted to the situation on the wards. For example, the intervention “know each other” was filled with different data at both wards. The ward culture plays an important role in the implementation of the intervention. This possibility of adaptation is explicitly described on the Safewards homepage. No changes were made to the model or the core of the interventions. Only the described possibilities for adaptation were used.

The next step was a call for expressions of interest regarding the contextual acceptance and the practical realization of the Safewards Model. This was carried out among all staff of the two locked wards. Staff members who were not willing to support the implementation (2 out of 40) agreed to change to another ward within the hospital. This was thought to ensure the actual implementation and a sustainable integration of the intervention “from within.”

The practical implementation process was led by the consultant psychiatrists and the ward managers. Supervision of the process was performed by the chief psychiatrist, the chief nurse, as well as ML and MS. Since each intervention was implemented within 1 month, this phase comprised of 10 months. The timely order of the implementation was decided upon the availability of the respective “expertise multipliers.” The implementation of each intervention was mainly carried out within regular weekly staff meetings. It included instructions on the interventions’ content, a demonstration of possibilities to transfer it into daily work routines, and the provision of working material. If necessary, the installation of items needed like posters, boxes, etc. was realized within that context as well. Staff who couldn’t attend the meetings were trained individually afterwards. Additionally, the Safewards steering group with project managers of each ward met once a week to prepare, discuss, and reflect on the implementation process. After all interventions were implemented, a time slot of 30 min in each weekly staff meeting had been scheduled for the Safewards Model. This time was used for reflecting upon experiences, success, and difficulties regarding the realization of the Safewards Model as well as for discussing its expansion.

The implementation process left enough space for each ward to shape according to their needs and their specific conditions and preferences. This procedure was chosen from the director of the department and the nursing director in order to enable the team to identify well with the Safewards Model.

### (3) Evaluation of the Implementation

The implementation of the Safewards Model was evaluated as part of a quality improvement initiative in the two locked wards of the department. Two members of the research team (JB and DJ) were responsible for compiling data. None of them were part of the clinical team or somehow else embedded in the implementation process. The study is a hybrid between an implementation study and an effectiveness study that is supposed to bring about scientific evidence on implementation challenges and outcomes as well as on the real-world effects of an evidence-based intervention (52). Sociodemographic (*age, sex, nationality*), disease-related (*main diagnosis*), and hospital-related (*ward*) data were collected from routine basic documentation for all patients who were exposed to coercive interventions. Furthermore, all coercive interventions that had been applied in these wards within 11 weeks before (t0) and 11 weeks after (t1) the implementation period of the Safewards Model were analyzed. Coercive interventions were defined as all actions taken against a patient’s will that limit his personal freedom or harm his physical integrity (53). They are only applied in emergency situations posing an acute risk of harm to self or others. At Vivantes Hospital Am Urban, three forms of coercive interventions—*mechanical restraint*, *forced medication*, and *limitation of freedom of movement*—as well as their combinations were applied and analyzed. *Mechanical restraint* (fixation) is defined as the use of a restrictive device to restrict the person’s free movement. In the respective locked wards, this device comprises of a set of limb cuffs and straps attached to a bed. Mechanical restraint is applied in emergency situations when no other measures to avoid harm for the person or for others including staff have been successful. *Forced medication* is defined as the involuntary administration of oral or intramuscular medication undertaken without the consent of the person being treated. It is only applied if either a) mechanical restraint was not enough to calm a patient down or he or she is (still) in danger to physically harm him- or herself, or b) a treatment order under the Berlin mental health act was made, or if c) a treatment order under the conditions of legal guardianship was made. In most cases, forced medication implies 5 to 10 min of physical restraint for administering the medication. *Limitation of freedom of movement* refers to the confinement of a patient in their room. In this time frame, he or she is allowed to leave the room only for specific purposes and for a limited time period. Limitation of freedom of movement is applied if patients are not able to keep the appropriate distance to other patients and to prevent patients form sensory overload, especially in manic phases. This form of containment has to be distinguished from “seclusion.” Seclusion is generally defined as the supervised confinement of a person alone in a room where the door cannot be opened from the inside. In the psychiatric inpatient units participating in our study, seclusion in a locked room was not applied.

We analyzed frequency and duration of the above stated three forms of coercive measures since previous empirical research showed and official recommendations suggested them to be practical measures of containment (33, 53–55).

For assessing fidelity to the Safewards Model, JB conducted the Organization Fidelity Checklist (32, 33) 4 to 8 months after the end of the implementation process. The checklist is a valid and reliable instrument for evaluating the quality and fidelity of 8 of the 10 Safewards interventions. It was applied in each ward separately. The Organization Fidelity Checklist reflects evidence that was on display, rather than the degree to which staff engaged with and used the displayed material (56). Criterions focusing on the use of an intervention in the ward (“0 = no”, “1 = yes”) were assessed regarding the interventions *Clear Mutual Expectations*, *Talk Down*, *Soft Words*, and *Discharge Messages*. Frequency of use was assessed regarding the interventions *Know Each Other* (“number of profiles: 1–≥10”), *Calm Down Methods* (“frequency of use: 1–≥10”), *Discharge Messages* (“number of discharge messages visible: 1–≥10”), *Mutual Help Meeting* (“number of meetings: 1–≥10”), and *Positive Words* (“number of handovers: 1–≥10”). Deviating from the original checklist, we did not count frequencies “since the last visit” but “since the end of the implementation process,” since fidelity was only checked once after the end of the implementation period.

### Statistical Analysis

The statistical plan was developed as basis for the evaluation before the implementation of the Safewards Model. Data analysis for descriptive statistics [frequency distribution (*n*), percentage distribution (%), mean (M), standard deviation (SD), and range] as well as for interferential statistics (chi-square test, unpaired *t* test, and Mann–Whitney test) was carried out using IBM SPSS Statistics 22. The quantification of the pre–post differences was determined by effect sizes (57). Benchmarks of coercive interventions (*percentage of patients exposed to coercive interventions*, *mean duration of coercive interventions*, *cumulative duration of coercive intervention per patient*, *average amount of coercive interventions per patient*, *duration of coercive interventions regarding the overall duration of stay*) were calculated according to official recommendations from the German Working group for the Prevention of Violence and Coercion in Psychiatry (12). Power calculation was not performed in advance due to a lack of solid data on coercive measures currently applied in acute psychiatry in Germany. Statistical significance was defined as *p* values of 5% or less.

## Results

### Implementation

All Safewards interventions were fully implemented in both wards. In ward B, the implementation was done according to the planned timeline. In ward A, the implementation process had to be paused for 8 months due to excessive workload and a major change in the team composition. Fidelity assessment after the implementation showed high model fidelity. The interventions *Clear Mutual Expectations* (“yes”), *Talk Down* (“yes”), *Soft Words* (“yes”), *Discharge Messages* (“yes,” “number of discharge messages visible: ≥10”), *Know Each Other* (“number of profiles: ≥10”), *Calm Down Methods* (“frequency of use: ≥10”), *Mutual Help Meeting* (“number of meetings: ≥10”), and *Positive Words* (“number of hand over: ≥10”) were fully implemented in both units according to the Safewards Model.

### Coercive Measures

Overall, in the two psychiatric wards, coercive interventions were performed on 250 occasions (ward A: *n*_t0_ = 79, *n*_t1_ = 93; ward B: *n*_t0_ = 57, *n*_t1_ = 21) in 103 patients (ward A: *n*_t0_ = 34, *n*_t1_ = 41; ward B: *n*_t0_ = 20, *n*_t1_ = 8) within the two study periods (t0 and t1). **Table 1** shows sociodemographic and disease-related data of patients that were exposed to coercive measures for each ward separately.

**Table 1:** Sociodemographic and disease-related data per ward with regard to patients that were exposed to coercive interventions before (t0) and after (t1) the implementation of the Safewards Model (*n*_ward A_ = 75; *n*_ward B_ = 28).

Variable	Variable label	Ward A *n* (%)		Ward B *n* (%)	
		t0	t1	t0	t1
Sex	Male	24 (70.6)	24 (58.5)	15 (75.0)	6 (75)
Nationality	German	30 (88.2)	33 (80.5)	18 (94.7)	8 (100)
Diagnosis group	F01 Organic, including symptomatic, mental disorders	1 (2.9)	−	−	−
	F1X Mental and behavioral disorders due to psychoactive substance use	10 (29.4)	10 (24.4)	6 (30.0)	1 (12.5)
	F2X Schizophrenia, schizotypal, and delusional disorders	15 (44.1)	20 (48.8)	11 (55.0)	5 (62.5)
	F3X Affective disorders	2 (5.9)	6 (14.6)	2 (10.0)	2 (25.0)
	F4X Neurotic, stress-related, and somatoform disorders	−	2 (4.9)	–	−
	F6X Disorders of adult personality and behavior	4 (11.8)	3 (7.3)	1 (5.0)	−
	F7X Mental retardation	1 (2.9)	−	−	−
	F9X Unspecified mental disorder	1 (2.9)	−	−	−

The age range of patients subjected to coercive interventions overall was 17 to 91 years [ward A: mean_case_: 37.6 ± 12.8 (*n* = 171); mean_patient_: 26.9 ± 14.2 (*n* = 74); ward B: mean_case_: 39.9 ± 8.4 (*n* = 78); mean_patient_: 35.6 ± 10.7 (*n* = 28)]. The term patient(-wise) refers to the number of patients who were exposed to coercive measures while the term case(-wise) refers to the number of coercive interventions that have been applied.

Patients exposed to coercive interventions before the implementation of the Safewards Model did not differ from patients who were subjected to these interventions afterwards regarding age, sex, nationality, and diagnosis group.

**Figure 1:**
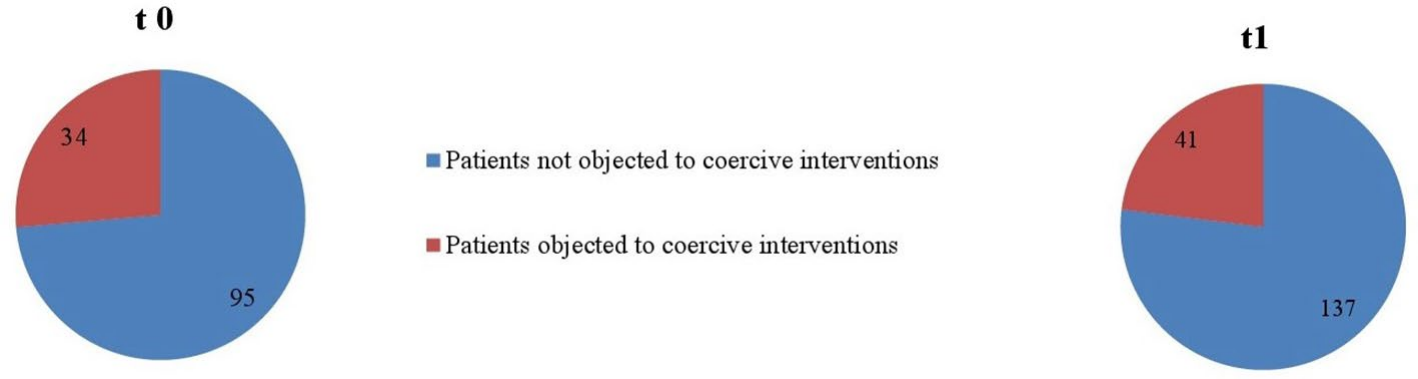
Number of patients objected to coercive interventions in relation to the overall number of patients in ward A (*n*_t0_ = 129, *n*_t1_ = 178).

**Figure 2:**
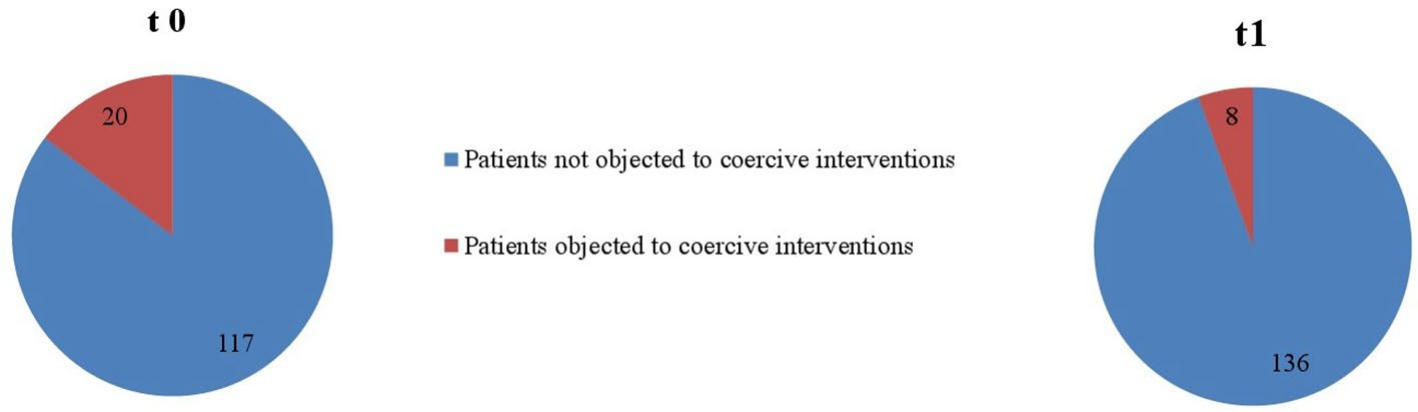
Number of patients objected to coercive interventions in relation to the overall number of patients in ward B (*n*_t0_ = 137, *n*_t1_ = 144).

**Figure 3:**
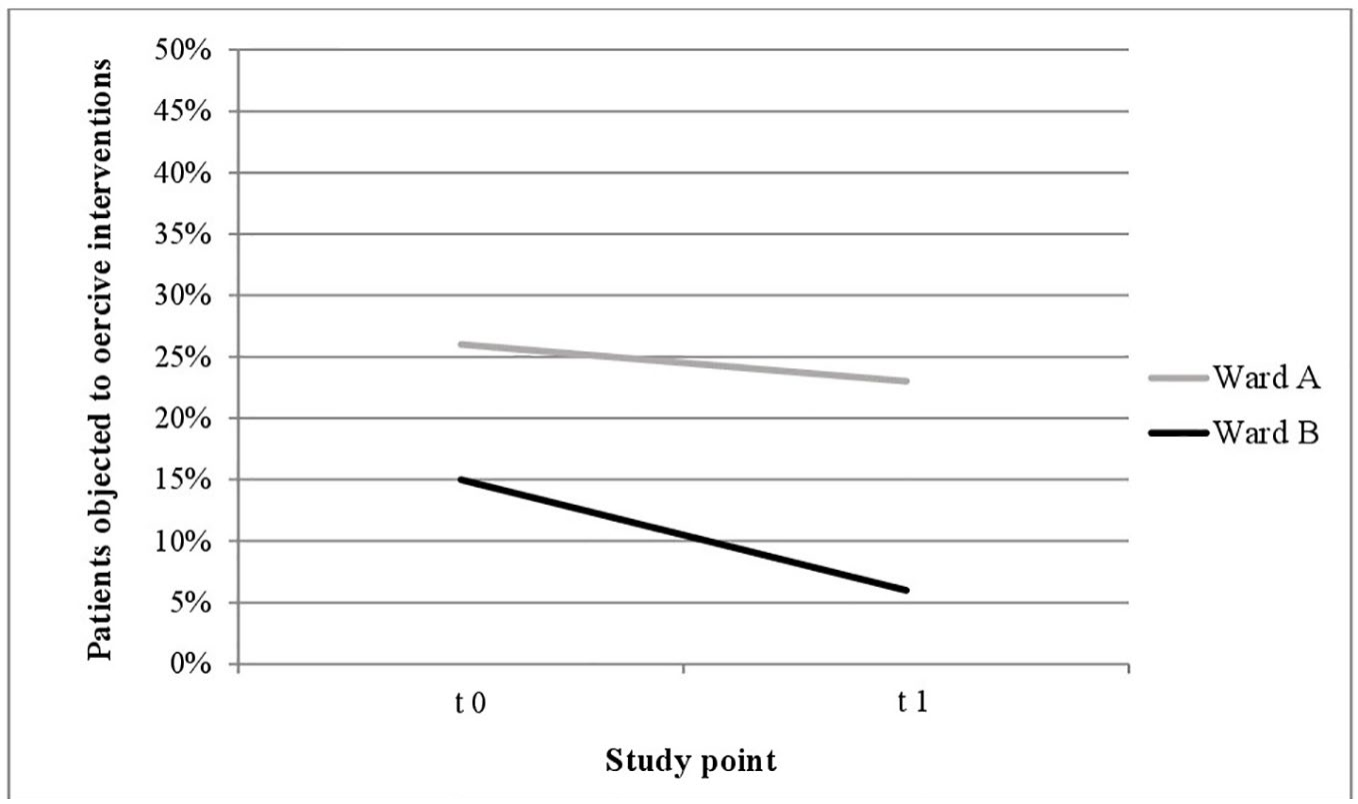
Descriptive change in coercive interventions (patient-wise) in relation to the overall number of patients in ward A (*n*_t0_ = 129, *n*_t1_ = 178) and ward B (*n*_t0_ = 137, *n*_t1_ = 144).

As seen in **Figures 1** and **2**, proportionally less people were exposed to coercive measures after the implementation of the Safewards Model in both wards with regard to the overall number of patients. However, the decrease was statistically significant only in ward B [χ^2^ (1, *n* = 281) = 6.40, *p* = 0.01]. **Figure 3** shows that there was no interaction between time and ward.

**Figure 4:**
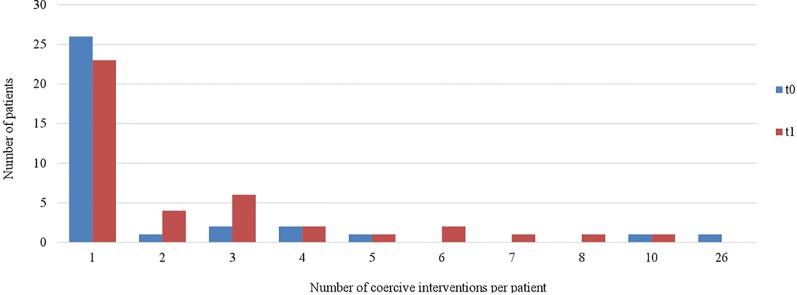
Number of coercive measures per patient in ward A (*n*_t0_ = 34, *n*_t1_ = 41).

**Figure 5:**
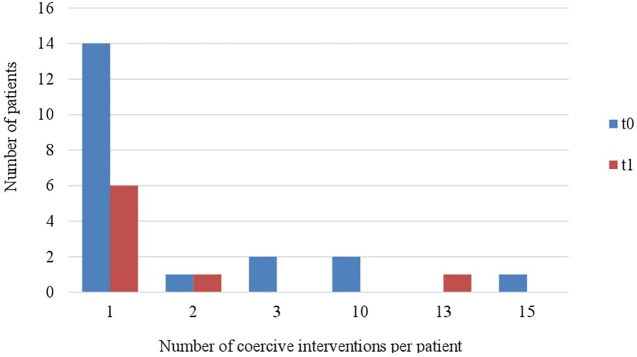
Number of coercive measures per patient in ward B (*n*_t0_ = 20, *n*_t1_ = 8).

**Figures 4** and **5** indicate that the range of all coercive interventions per patient decreased in both wards (ward A: range_t0_ = 1–26, range_t1_ = 1–10; ward B: range_t0_ = 1–15, range_t1_ = 1–13). Furthermore, it shows that fewer patients were exposed to multiple occasions of coercive measures after the implementation of the Safewards Model.

**Figure 6:**
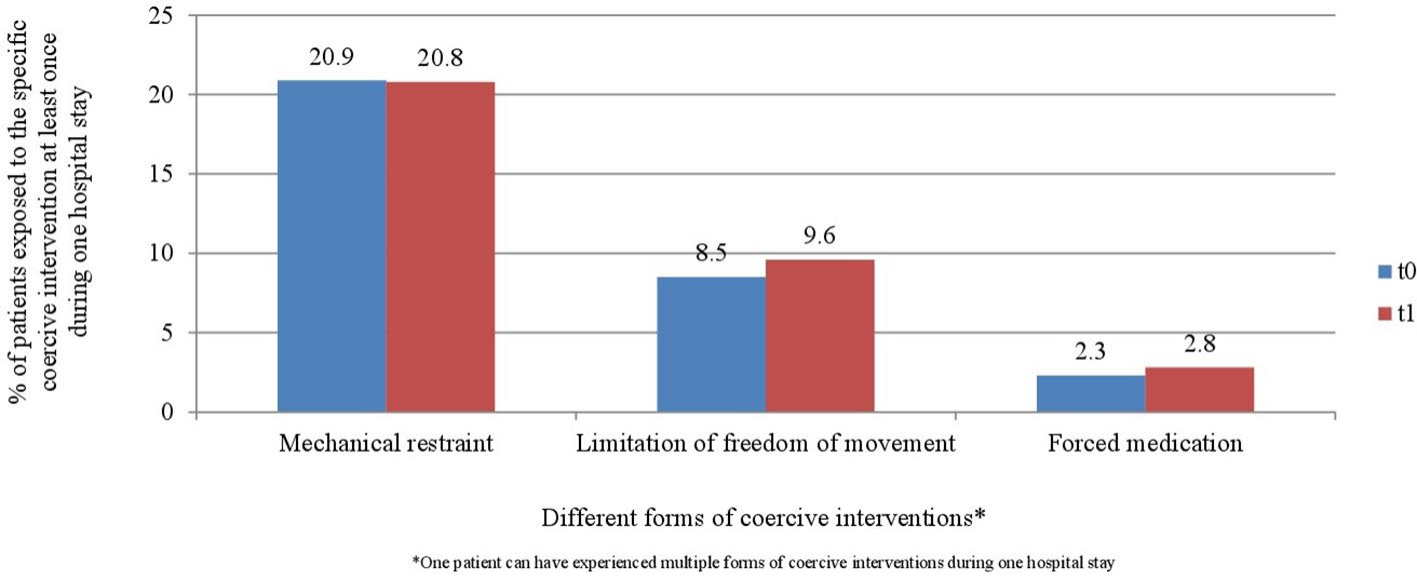
Percentage of patients exposed to the specific methods of coercive interventions in relation to the overall number of patients before and after the implementation of the Safewards model in ward A (*n*_t0_ = 129, *n*_t1_ = 178).

**Figure 7:**
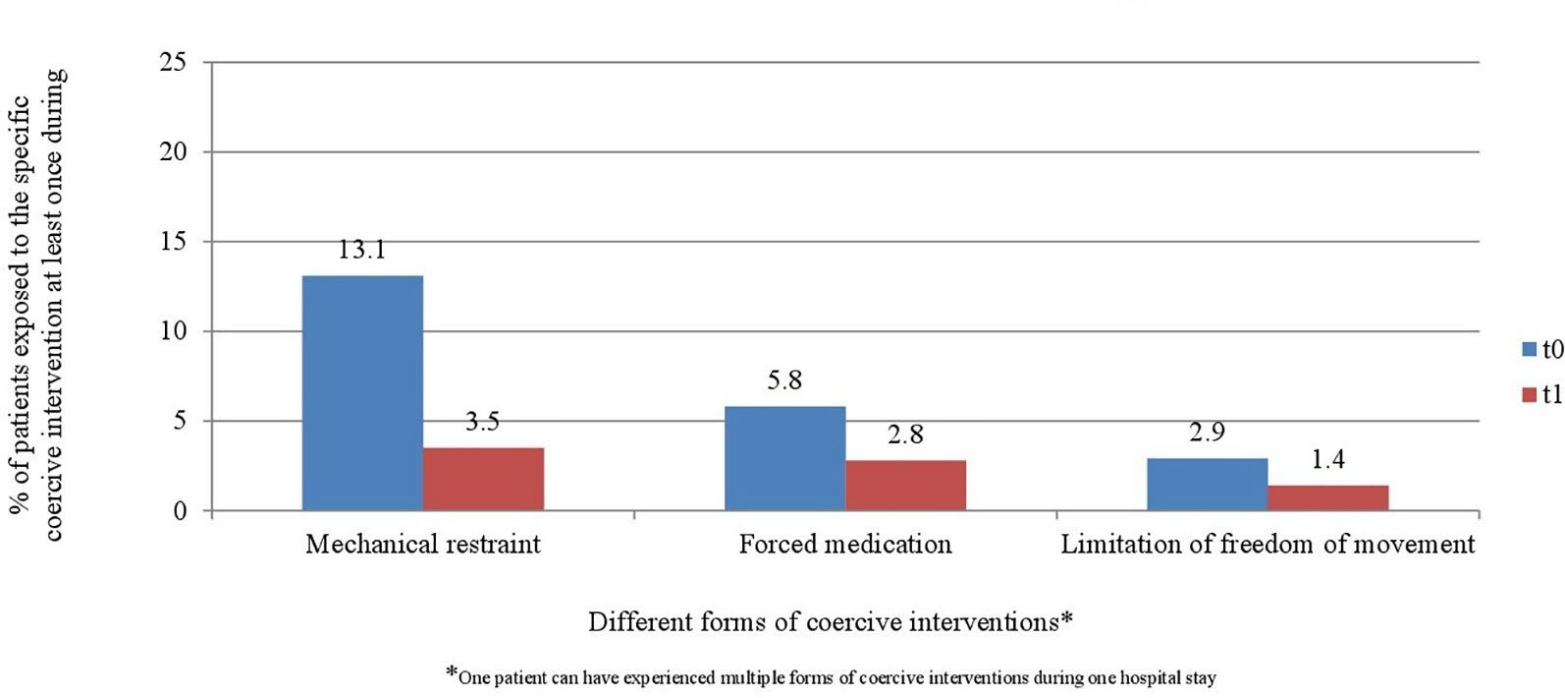
Percentage of patients exposed to the specific methods of coercive interventions in relation to the overall number of patients before and after the implementation of the Safewards model in ward B (*n*_t0_ = 137, *n*_t1_ = 144).

**Figures 6** and **7** display the percentage of patients exposed to the specific methods of coercive interventions at least once during their hospital stay in relation to the overall number of patients analyzed for each ward separately. Herby, one patient can have experienced multiple forms of interventions.

Results relating to duration of coercive interventions (case-wise) for each ward separately are displayed in **Table 2**. As seen there, the duration of coercive interventions overall decreased case-wise in ward B significantly after the implementation of the Safewards Model.

**Table 2:** Duration of coercive interventions per case separated by ward before (t0) and after (t1) the implementation of the Safewards Model (reported in hours).

Variable^1,2^	t0				t1				*U* value	*p* value	ES^2^	95% CI
	*n*	Range	Mean	SD	*n*	Range	M	SD				
**Coercive interventions overall**	132	0.08–1860.82	64.7	273.80	113	0.08–953.73	28.7	105.16	0.673	0.673	−0.169	−0.42, 0.083
Ward A	77	0.17–1860.82	92.2	348.32	92	0.08–953.73	34.3	115.86	−0.537	0.591	−0.232	−0.535, 0.072
Ward B	55	0.08–648	26.2	92.30	21	0.08–31.23	3.9	7.67	−2.142	0.032	−0.282	−0.787, 0.222
**Mechanical restraint**	80	0.17–43.75	6.5	7.70	60	0.08–36.5	6.3	7.98	−0.809	0.419	−0.026	−0.361, 0.309
Ward A	62	0.17–43.75	6.8	7.92	57	0.08–36.5	6.5	8.11	−0.902	0.367	−0.037	−0.397, 0.322
Ward B	18	0.17–28	5.6	7.00	3	0.67–5.67	2.4	2.86	−1.257	0.209	−0.479	−1.709, 0.752
**Forced medication**	4	0.08–0.17	0.2	0.04	1	0.08	.08	–	−1.225	0.221	–	–
Ward A	1	0.17	0.2	–	–	–	–	–	–	–	–	–
Ward B	3	0.08–0.17	0.1	0.05	1	0.08	.08	–	−1.000	0.317	–	–
**Forced medication and mechanical restraint**	17	0.08–6	1.2	1.62	19	0.08–10.75	1.5	2.75	−0.736	0.462	0.131	−0.524, 0.786
Ward A	2	0.17–0.83	0.5	0.47	5	0.08–5.83	2.5	2.15	−1.162	0.245	1.034	−0.693, 2.761
Ward B	15	0.08–6	1.3	1.71	14	0.08–10.75	1.2	2.92	−0.458	0.647	−0.042	−0.771, 0.686
**Forced medication and limitation of freedom of movement** (Ward A)	–	–	–	–	1	–	1.4	–	–	–	–	–
**Limitation of freedom of movement**	30	0.5–1860.8	265.6	533.33	32	0.42–953.73	88.4	186.22	−0.211	0.833	−0.427	−0.93, 0.077
Ward A	11	6–1860.8	604.0	765.16	29	0.42–953.73	95.5	194.47	−1.895	0.058	−1.192	−1.934, −0.451
Ward B	19	0.5–648	69.6	149.89	3	10. 98–31.23	19.2	10.68	0.886	0.929	−0.354	−1.576, 0.868

^1^Cases lacking information of duration of coercive intervention (n = 2) and cases with implausible values (n = 3) were excluded from the analysis regarding duration of coercive interventions. ^2^Interpretation of the effect sizes according to Cohen (57): d = 0.2 to d = 0.4: small effect, d = 0.5 to d = 0.7: medium effect, d ≥ 0.8: large effect.

On average, patients exposed to coercive interventions experienced these interventions during their hospital stay in ward A 2.33 times before and 2.27 times after and in ward B 2.85 times before and 2.63 times after the implementation of the Safewards Model. Total duration of coercive interventions in relation to the overall duration of the hospital inpatient stay decreased in ward A from 17% before to 12% and in ward B from 7% to 1% after the implementation of the Safewards Model. Coefficients regarding the cumulative duration of coercive interventions per patient, another important benchmark of coercive interventions, are displayed in **Table 3**.

**Table 3:** Cumulative duration of coercive interventions per patient separated by ward before (t0) and after (t1) the implementation of the Safewards Model (reported in hours).

Variable^1^	t0			t1			*U* value	*p* value	ES^2^	95% CI
	*n*	Mean	SD	*n*	Mean	SD				
**Coercive interventions overall**	51	167.52	461.86	49	66.07	168.35	−0.100	0.920	−0.29	− 0.684, 0.105
Ward A	33	215.17	539.99	41	76.99	182.31	−0.098	0.922	−0.359	−0.821, 0.103
Ward B	18	80.17	256.97	8	10.14	12.02	−0.556	0.579	−0.324	−1.161, 0.514
**Mechanical restraint**	38	13.75	25.06	35	10.85	17.06	−0.806	0.420	−0.134	−0.594, 0.325
Ward A	25	16.84	30.16	32	11.64	17.63	−0.667	0.504	−0.217	−0.742, 0.307
Ward B	13	7.80	7.67	3	2.36	2.86	0.139	0.139	−0.756	−2.038, 0.527
**Forced medication**	3	0.2	0.05	1	0.08	–	0.157	0.157	–	–
Ward A	1	0.17	–	–	–	–	–	–	–	–
Ward B	2	0.21	0.06	1	0.08	–	−1.225	0.221	–	–
**Forced medication and mechanical restraint**	9	2.21	2.57	8	3.60	3.36	−1.155	0.248	0.469	−0.496, 1.435
Ward A	2	0.5	0.47	5	2.47	2.15	−1.162	0.245	1.017	−0.707, 2.741
Ward B	7	2.7	2.74	3	5.5	4.65	−1.254	0.210	0.842	−0.56, 2.244
**Forced medication and limitation of freedom of movement** (Ward A)	–	–	–	1	1.42	–		–	–	–
**Limitation of freedom of movement**	14	569.05	709.93	18	157.09	239.85	−1.767	0.077	−0.821	−1.548, −0.095
Ward A	10	664.42	778.60	16	173.13	250.45	−1.503	0.133	−0.952	−1.783, −0.12
Ward B	4	330.60	509.12	2	28.73	3.54	−1.852	0.064	−0.685	−2.426, 1.056
*^1^* *Cases lacking information of duration of coercive interventions (n = 2) and cases with implausible values (n = 3) were excluded from the analysis regarding duration of methods of coercive interventions.* *^2^* *Interpretation of the effect sizes according to Cohen (57): d = 0.2 to d = 0.4: small effect, d = 0.5 to d = 0.7: medium effect, d ≥ 0.8: large effect.*										

## Discussion

This is the first study that evaluated the implementation of the Safewards Model in locked acute psychiatric wards in the German health care context with regard to coercive measures. The characteristics of the patient population regarding age range and distribution are in line with numerous other surveys, e.g., a study by Adorjan et al. that evaluated the use of coercive measures in eight German psychiatric hospitals (58). Other studies also found coercive measures to be most commonly used in patients with schizophrenia (59) followed by patients with substance use disorders (58, 60). A further German study found patients with an organic mental disorder to be most commonly exposed to coercive measures followed by patients with a schizophrenic disorder (12). However, this study is only partially comparable to ours since patients with organic disorders at Vivantes Hospital Am Urban are usually admitted to a special ward for older people with mental health problems. In line with other studies, most patients in our study experienced coercive interventions only once during their hospital stay, while the range of occasions of coercive interventions per patient was rather small (59). Overall, it can be assumed that the use of coercive interventions presented in our study can be generalized to other German psychiatric hospitals.

The quality of the implementation showed high fidelity to the Safewards Model. High fidelity was also found in other studies outlining positive results of the implementation of the Safewards Model regarding the reduction of coercive interventions (32, 33). At the same time, it must be considered that the fidelity checklist evaluates only the objective, visible evidence of the application of the Safewards Model. It does not indicate the degree to which staff is engaged with the principles of the model or rather the staff’s attitude toward the Safewards Model. Thus, fidelity outcomes do not indicate whether and to what extent staff members have internalized the overall idea of the Safewards Model. They also do not show if staff is successfully applying it in everyday work routine.

The fact that results regarding frequency and duration of coercive measures differ substantially between wards underlines these aspects. This phenomenon is well-known from previous reports where engagement with the Safewards Model varies between wards and even within wards (2). Reasons discussed for those differences on the level of wards are number, education, resilience, professional self-conception, professional experience, stress level, and personality of employees (59). In our study, there are several aspects in both wards that might contribute to the differences in results. In ward A, e.g., there had been a change of the consultant psychiatrist during the implementation period. This might have led to uncertainty within the team regarding several care aspects such as—among others—the application of coercive measures. Further uncertainty was brought into the team through high staff turnover during the implementation period. The unexpected and sudden termination of the head nurse resulted in a longer period of vacancy and team guidance and affected the team and its care routines. Additionally, age and professional experience of staff in ward A was lower than that in ward B. These circumstances as well as the lack of additional time or staff resources for the implementation resulted in a disruption of the process in ward A for 8 months. The aspects mentioned above—high staff turnover, little professional experience, lack of additional resources—can be regarded as barriers for the implementation process. On the other hand, there were several circumstances in ward B that might have facilitated the implementation of the Safewards Model and the reduction of the use of coercive measures there. First of all, ward B was led by one and the same consultant psychiatrist for several years—a fact that enhanced stability and routine in the team. Furthermore, this psychiatrist was engaged in the reduction of coercive measures for a long time through other contexts. Additionally, the team had hardly any turnover for a long period of time and was familiar to applying standards in dealing with conflicts and containment. One change of staff that occurred before the implementation of the Safewards Model—the change of the head nurse—had been prepared for several years and thus brought hardly any uncertainty or disturbances into the team. The outlined aspects—stable staffing, higher professional experience, stringent policy of dealing with conflict, and containment over a longer time—can be regarded as facilitators of the implementation process. For further studies, named variables should be included into the evaluation of the implementation in order to gain further insight into Safewards implementation success factors.

We found that the amount of patients exposed to coercive interventions in relation to the overall number of patients—the most important indicator for coercive interventions—as well as the mean duration of coercive interventions were significantly lower after the implementation of the Safewards Model. Furthermore, we found a decrease in the range of coercive interventions per patient, in the number of coercive measures per patient, and in the total time spent under coercive circumstances in relation to the overall duration of the hospital inpatient stay after the implementation of the Safewards Model. These results are in line with outcomes found in a randomized controlled trial that investigated the implementation of the Safewards Model (32). Furthermore, our results are similar to those of studies that evaluated interventions focusing on de-escalation and anti-aggression staff training aiming at reducing coercive interventions (16).

There are some studies that did not find positive changes in coercive measures after the implementation of the Safewards Model (36) or other methods aiming at reducing coercive interventions in hospitals (55). In the study of Price et al. (36), however, contrary to ours, staff acceptance and adherence to the intervention were low. One reason for the positive changes found in our study could be the fact that staff opted for the implementation of the Safewards Model. Thus, only motivated staff members supporting the model were present at follow-up. Nevertheless, we did not find a significant reduction in the frequency of forced medication as opposed to a large retrospective register study (13). Furthermore, relatively wide confidence intervals suggested varying success with implementation of the Safewards Model (32). In order to consolidate and expand positive changes after implementing the Safewards Model, staff should reflect upon their experience with the interventions and refresh its contents regularly. This could be realized, e.g., within team meetings, supervision, and appropriate training (61). Further important aspects for a sustainable reduction of coercive interventions were found to be adequate staffing and low staff turnover (3). Time and material resources are further aspects needed to successfully implement alternative methods for reducing conflict and containment (3). Regular assessment of frequency and duration of coercive interventions through routine data over time would give insight if efforts undertaken are effective and sustainable.

Results are presented with regard to each coercive intervention separately for outlining the large differences in frequency and duration of the use of the different kinds of coercive interventions. This mode of presentation is in line with recommendations as well as other studies on coercion (13, 33, 59, 62). Our mode of presentation differs from studies that presented results separately for each diagnostic group (12). To our point of view, this would not have been reasonable for this study because most diagnostic groups comprised only a small number of patients.

Comparable to other studies, mechanical restraint was the most commonly used form of coercive measure in our study (58). While the average number of coercive interventions per patient in our study was lower, the mean duration and the cumulative duration of coercive interventions overall were higher than in another German study (62). These differences may be explained by the long duration of limitation of freedom of movement in the study hospital. In contrast to seclusion, this milder method of containment can be applied over a longer period and thus biases sum cores on the overall duration of coercive interventions. The study wards had no locked rooms, and thus no seclusion, but only arrangements to stay in the patient room for a certain time period were enforced. The named differences could also be explained by the fact that we evaluated coercive interventions in two acute wards in one hospital and did not look at wards in different psychiatric hospitals. It is known, however, that clinical factors, such as high levels of psychotic symptoms and high levels of perceived coercion at admission are discussed as being associated with the use of coercive measures (4). The heterogeneous databases of other studies could also explain the comparatively higher number of patients exposed to coercive interventions in relation to the overall number of patients in our study (55, 58, 62). Another explanation would be differences in documentation between hospitals (59). To underpin our findings and check for their sustainability, our study needs to be repeated within a controlled study design with more participants and over a longer period. Since the implementation of the Safewards Model has positive effects in different health systems, it is a promising approach for the reduction of coercive measures in acute psychiatry.

### Limitations

The study has several limitations. Since the Safewards Model was implemented in both acute mental health wards during the same time period, i.e., the whole acute sector of the hospital, with joint workshops as part of a hospital-wide approach, randomization and a control group design were not possible. A control group would have resulted in the Safewards Model being implemented in one ward 1 year later. With a pre–post study, we did not have control over other elements possibly affecting the outcomes. Therefore, inferences must be drawn with caution, and changes might not be fully attributed to the intervention. Results might be biased due to a change of staff members during the evaluation period and an implementation pause in one ward. Furthermore, data on coercive interventions were only gathered over a period of 11 weeks, which might have biased the results due to seasonal fluctuations regarding the number of patients admitted to the wards. Nevertheless, this is the first study that evaluates the implementation of the Safewards Model in acute inpatient psychiatry or rather in locked wards in Germany. It provides evidence of positive effects regarding the reduction of coercive interventions. Our study results add to the evidence base of the Safewards Model as a complex intervention that applies some of the six core strategies (6CS) identified by the US National Association of State Mental Health Program Directors Medical Directors Council (63). These account as critical elements of success to reduce restraint and seclusion in mental health care. Safewards, 6CS, and other complex approaches aim at building a more therapeutic environment with outcomes according to intervention fidelity and facility or ward characteristics and patterns (64).

## Ethics Statement

An ethics approval for the study conducted was not required. The Department of Psychiatry, Psychotherapy and Psychosomatic Medicine at Vivantes Hospital Am Urban in Berlin is obligated by German law to record all coercive measures conducted regarding sort and duration. Once a year, the hospital reports these data to Berlin senate (“Senatsabfrage der Zahlen von Unterbringung und Sicherungsmaßnahmen”). In this manuscript, we exclusively analyzed data that had been collected for this purpose. The acquisition of data regarding coercive measures does not affect or compromise any patients and does not include patient contact at all, because it is recorded in corresponding hospital documents by hospital staff right after the coercive measure was carried out.

## Author Contributions

All authors listed have made substantial, direct, and intellectual contribution to the work and approved it for publication.

## Conflict of Interest Statement

The authors declare that the research was conducted in the absence of any commercial or financial relationships that could be construed as a potential conflict of interest.

The handling editor declared a shared affiliation, though no current collaboration, with the author SW at the time of the review.
