# Corrigendum: Preventing and Reducing Coercive Measures—An Evaluation of the Implementation of the Safewards Model in Two Locked Wards in Germany

**DOI:** 10.3389/fpsyt.2020.00162

**Published:** 2020-03-11

**Authors:** Johanna Baumgardt, Dorothea Jäckel, Heike Helber-Böhlen, Nicole Stiehm, Karin Morgenstern, Andre Voigt, Enrico Schöppe, Ann-Kathrin Mc Cutcheon, Edwin Emilio Velasquez Lecca, Michael Löhr, Michael Schulz, Andreas Bechdolf, Stefan Weinmann

**Affiliations:** ^1^Department of Psychiatry, Psychotherapy and Psychosomatic Medicine, Vivantes Hospital Am Urban und Vivantes Hospital im Friedrichshain, Charité–Universitätsmedizin Berlin, Berlin, Germany; ^2^Department of Psychiatry and Psychotherapy, Center for Psychosocial Medicine, University Medical Center Hamburg-Eppendorf, Hamburg, Germany; ^3^Landschaftsverband Westfalen-Lippe, Hospital Gütersloh, Gütersloh, Germany; ^4^Diakonie University of Applied Sciences, Bielefeld, Germany; ^5^ORYGEN, National Center of Excellence of Youth Mental Health, University of Melbourne, Melbourne, VIC, Australia; ^6^Department for Psychiatry and Psychotherapy, University Hospital Cologne, Cologne, Germany; ^7^University Psychiatric Hospital Basel, Basel, Switzerland

**Keywords:** Safewards Model, conflict, coercive measures, acute psychiatric care, inpatient treatment, locked ward

In the original article, there was an error. In the article it says we analyzed a period of 10 weeks before and after the implementation of the Safewards model. Actually, we analyzed a period of 11 weeks. Furthermore, in the article it says we compared the amount of patients exposed to coercive interventions with patients admitted to the same ward at the same time. Actually, we compared patients exposed to coercive interventions with the overall amount of patients staying at the same time. Additionally, the amount of patients overall was incorrect due to inaccurate information from our in-house hospital system, which we were only recently informed about. This only affected analysis or ratios where we compared patients exposed to coercive measures with patients who were not exposed to coercive measures. However, the direction of the effects stayed the same (=with regard to the overall number of patients proportionally less people were exposed to coercive measures after the implementation of the Safewards Model in both wards) with a statistically significant effect only in ward B.

The fully corrected paragraphs are below:

The **Abstract**, subsection **Materials and Methods**:

“We evaluated outcomes of the implementation of the Safewards Model in two locked psychiatric wards in Germany. Frequency and duration of coercive interventions applied during a period of 11 weeks before and 11 weeks after the implementation period were assessed through routine data. Fidelity to the Safewards Model was assessed by the Organization Fidelity Checklist.”

The **Abstract**, subsection **Results**:

“Fidelity to the Safewards Model was high in both wards. The overall use of coercive measures differed significantly between wards [case-wise: χ^2^ (1, *n* = 250) = 35.34, *p* ≤ 0.001; patient-wise: χ^2^ (1, *n* = 103) = 21.45, *p* ≤ 0.001] and decreased post-implementation. In one ward, the number of patients exposed to coercive interventions in relation to the overall number of patients decreased significantly [χ^2^ (1, 281) = 6.40, *p* = 0.01]. Furthermore, the mean duration of coercive interventions overall declined significantly [U(55,21) = −2.142, *p* = 0.032] with an effect size of Cohen's *d* = −0.282 (95% CI: −0.787, 0.222) in that ward. Both aspects declined as well in the other ward, but not significantly.”

The **Materials and Methods** section, subsection **(3) Evaluation of the Implementation**, paragraph 1:

“The implementation of the Safewards Model was evaluated as part of a quality improvement initiative in the two locked wards of the department. Two members of the research team (JB and DJ) were responsible for compiling data. None of them were part of the clinical team or somehow else embedded in the implementation process. The study is a hybrid between an implementation study and an effectiveness study that is supposed to bring about scientific evidence on implementation challenges and outcomes as well as on the real-world effects of an evidence-based intervention (52). Sociodemographic (*age, sex, nationality*), disease-related (*main diagnosis*), and hospital-related (*ward*) data were collected from routine basic documentation for all patients who were exposed to coercive interventions. Furthermore, all coercive interventions that had been applied in these wards within 11 weeks before (t0) and 11 weeks after (t1) the implementation period of the Safewards Model were analyzed. Coercive interventions were defined as all actions taken against a patient's will that limit his personal freedom or harm his physical integrity (53). They are only applied in emergency situations posing an acute risk of harm to self or others. At Vivantes Hospital Am Urban, three forms of coercive interventions—*mechanical restraint, forced medication*, and *limitation of freedom of movement*—as well as their combinations were applied and analyzed. *Mechanical restraint* (fixation) is defined as the use of a restrictive device to restrict the person's free movement. In the respective locked wards, this device comprises of a set of limb cuffs and straps attached to a bed. Mechanical restraint is applied in emergency situations when no other measures to avoid harm for the person or for others including staff have been successful. *Forced medication* is defined as the involuntary administration of oral or intramuscular medication undertaken without the consent of the person being treated. It is only applied if either a) mechanical restraint was not enough to calm a patient down or he or she is (still) in danger to physically harm him- or herself, or b) a treatment order under the Berlin mental health act was made, or if c) a treatment order under the conditions of legal guardianship was made. In most cases, forced medication implies 5 to 10 min of physical restraint for administering the medication. *Limitation of freedom of movement* refers to the confinement of a patient in their room. In this time frame, he or she is allowed to leave the room only for specific purposes and for a limited time period. Limitation of freedom of movement is applied if patients are not able to keep the appropriate distance to other patients and to prevent patients form sensory overload, especially in manic phases. This form of containment has to be distinguished from “seclusion.” Seclusion is generally defined as the supervised confinement of a person alone in a room where the door cannot be opened from the inside. In the psychiatric inpatient units participating in our study, seclusion in a locked room was not applied.”

The **Materials and Methods** section, subsection **Statistical Analysis**:

“The statistical plan was developed as basis for the evaluation before the implementation of the Safewards Model. Data analysis for descriptive statistics [frequency distribution (*n*), percentage distribution (%), mean (M), standard deviation (SD), and range] as well as for interferential statistics (chi-square test, unpaired *t* test, and Mann–Whitney test) was carried out using IBM SPSS Statistics 22. The quantification of the pre–post differences was determined by effect sizes (57). Benchmarks of coercive interventions (*percentage of patients exposed to coercive interventions, mean duration of coercive interventions, cumulative duration of coercive intervention per patient, average amount of coercive interventions per patient, duration of coercive interventions regarding the overall duration of stay*) were calculated according to official recommendations from the German Working group for the Prevention of Violence and Coercion in Psychiatry (12). Power calculation was not performed in advance due to a lack of solid data on coercive measures currently applied in acute psychiatry in Germany. Statistical significance was defined as *p* values of 5% or less.”

The **Results** section, subsection **Coercive Measures**, paragraphs 1, 4, and 6:

“Overall, in the two psychiatric wards, coercive interventions were performed on 250 occasions (ward A: *n*_t0_ = 79, *n*_t1_ = 93; ward B: *n*_t0_ = 57, *n*_t1_ = 21) in 103 patients (ward A: *n*_t0_ = 34, *n*_t1_ = 41; ward B: *n*_t0_ = 20, *n*_t1_ = 8) within the two study periods (t0 and t1). Table 1 shows sociodemographic and disease-related data of patients that were exposed to coercive measures for each ward separately.”

“As seen in Figures 1 and 2, proportionally less people were exposed to coercive measures after the implementation of the Safewards Model in both wards with regard to the overall number of patients. However, the decrease was statistically significant only in ward B [χ^2^ (1, *n* = 281) = 6.40, *p* = 0.01]. Figure 3 shows that there was no interaction between time and ward.”

“Figures 6 and 7 display the percentage of patients exposed to the specific methods of coercive interventions at least once during their hospital stay in relation to the overall number of patients analyzed for each ward separately. Herby, one patient can have experienced multiple forms of interventions.”

The **Discussion** section, paragraphs 4 and 7:

“We found that the amount of patients exposed to coercive interventions in relation to the overall number of patients—the most important indicator for coercive interventions—as well as the mean duration of coercive interventions were significantly lower after the implementation of the Safewards Model. Furthermore, we found a decrease in the range of coercive interventions per patient, in the number of coercive measures per patient, and in the total time spent under coercive circumstances in relation to the overall duration of the hospital inpatient stay after the implementation of the Safewards Model. These results are in line with outcomes found in a randomized controlled trial that investigated the implementation of the Safewards Model (32). Furthermore, our results are similar to those of studies that evaluated interventions focusing on de-escalation and anti-aggression staff training aiming at reducing coercive interventions (16).”

“Comparable to other studies, mechanical restraint was the most commonly used form of coercive measure in our study (58). While the average number of coercive interventions per patient in our study was lower, the mean duration and the cumulative duration of coercive interventions overall were higher than in another German study (62). These differences may be explained by the long duration of limitation of freedom of movement in the study hospital. In contrast to seclusion, this milder method of containment can be applied over a longer period and thus biases sum cores on the overall duration of coercive interventions. The study wards had no locked rooms, and thus no seclusion, but only arrangements to stay in the patient room for a certain time period were enforced. The named differences could also be explained by the fact that we evaluated coercive interventions in two acute wards in one hospital and did not look at wards in different psychiatric hospitals. It is known, however, that clinical factors, such as high levels of psychotic symptoms and high levels of perceived coercion at admission are discussed as being associated with the use of coercive measures (4). The heterogeneous databases of other studies could also explain the comparatively higher number of patients exposed to coercive interventions in relation to the overall number of patients in our study (55, 58, 62). Another explanation would be differences in documentation between hospitals (59). To underpin our findings and check for their sustainability, our study needs to be repeated within a controlled study design with more participants and over a longer period. Since the implementation of the Safewards Model has positive effects in different health systems, it is a promising approach for the reduction of coercive measures in acute psychiatry.”

The **Discussion** section, subsection **Limitations**:

“The study has several limitations. Since the Safewards Model was implemented in both acute mental health wards during the same time period, i.e., the whole acute sector of the hospital, with joint workshops as part of a hospital-wide approach, randomization and a control group design were not possible. A control group would have resulted in the Safewards Model being implemented in one ward 1 year later. With a pre–post study, we did not have control over other elements possibly affecting the outcomes. Therefore, inferences must be drawn with caution, and changes might not be fully attributed to the intervention. Results might be biased due to a change of staff members during the evaluation period and an implementation pause in one ward. Furthermore, data on coercive interventions were only gathered over a period of 11 weeks, which might have biased the results due to seasonal fluctuations regarding the number of patients admitted to the wards. Nevertheless, this is the first study that evaluates the implementation of the Safewards Model in acute inpatient psychiatry or rather in locked wards in Germany. It provides evidence of positive effects regarding the reduction of coercive interventions. Our study results add to the evidence base of the Safewards Model as a complex intervention that applies some of the six core strategies (6CS) identified by the US National Association of State Mental Health Program Directors Medical Directors Council (63). These account as critical elements of success to reduce restraint and seclusion in mental health care. Safewards, 6CS, and other complex approaches aim at building a more therapeutic environment with outcomes according to intervention fidelity and facility or ward characteristics and patterns (64).”

Furthermore, the corrected Figures 1–3, 6, 7 and their legends appear below. For Figures 6, 7, we were told that the demonstration of “combined” forms of coercive interventions in addition to the “single” forms of coercive interventions (mechanical restraint - forced medication - limitation of freedom of movement) is confusing to the reader. Therefore, we split the “combined” forms and added it to the corresponding “single” form of coercive intervention.

**Figure 1:**
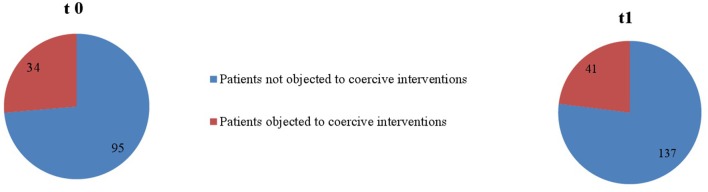
Number of patients objected to coercive interventions in relation to the overall number of patients in ward A (*n*_t0_ = 129, *n*_t1_ = 178).

**Figure 2:**
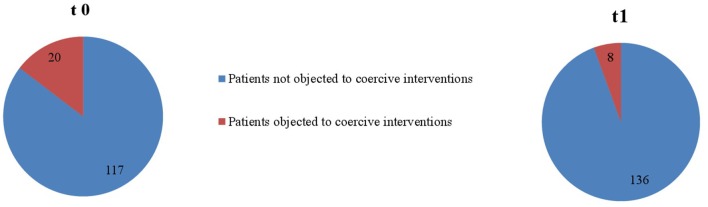
Number of patients objected to coercive interventions in relation to the overall number of patients in ward B (*n*_t0_ = 137, *n*_t1_ = 144).

**Figure 3:**
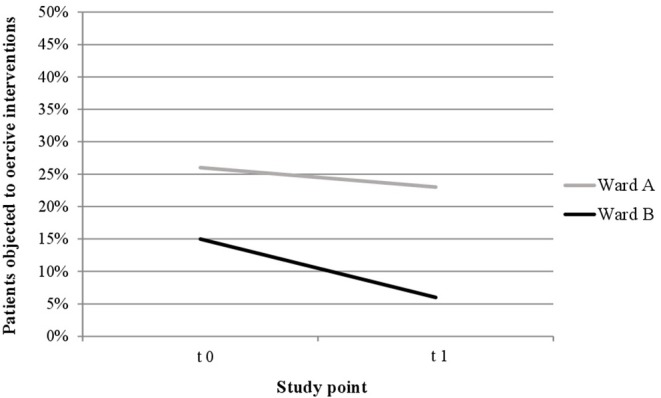
Descriptive change in coercive interventions (patient-wise) in relation to the overall number of patients in ward A (*n*_t0_ = 129, *n*_t1_ = 178) and ward B (*n*_t0_ = 137, *n*_t1_ = 144).

**Figure 6:**
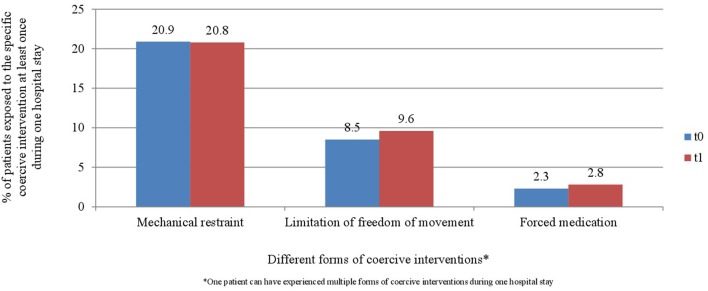
Percentage of patients exposed to the specific methods of coercive interventions in relation to the overall number of patients before and after the implementation of the Safewards model in ward A (*n*_t0_ = 129, *n*_t1_ = 178).

**Figure 7:**
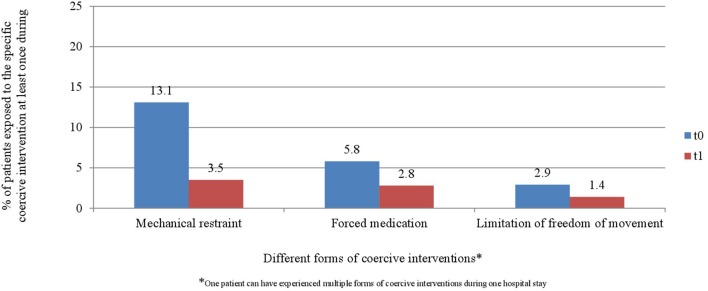
Percentage of patients exposed to the specific methods of coercive interventions in relation to the overall number of patients before and after the implementation of the Safewards model in ward B (*n*_t0_ = 137, *n*_t1_ = 144).

The authors apologize for these errors and state that this does not change the scientific conclusions of the article in any way. The original article has been updated.

